# Volume of Interest Analysis of Spatially Normalized PRESTO Imaging to Differentiate between Parkinson Disease and Atypical Parkinsonian Syndrome

**DOI:** 10.2463/mrms.mp.2015-0132

**Published:** 2016-03-21

**Authors:** Keita Sakurai, Etsuko Imabayashi, Aya M. Tokumaru, Kimiteru Ito, Keigo Shimoji, Motoo Nakagawa, Yoshiyuki Ozawa, Masashi Shimohira, Masaki Ogawa, Satoru Morimoto, Ikuko Aiba, Noriyuki Matsukawa, Yuta Shibamoto

**Affiliations:** 1 Department of Radiology, Nagoya City University Graduate School of Medical Sciences, 1 Kawasumi, Mizuho-cho, Mizuho-ku, Nagoya 467-8601, Japan; 2 Integrative Brain Imaging Center, National Center of Neurology and Psychiatry; 3 Department of Diagnostic Radiology, Tokyo Metropolitan Medical Center of Gerontology; 4 Department of Neurology, Tokyo Metropolitan Geriatric Hospital; 5 Department of Neurology, National Hospital Organization Higashi Nagoya National Hospital; 6 Department of Neurology and Neuroscience, Nagoya City University Graduate School of Medical Sciences

**Keywords:** multiple system atrophy (MSA), progressive supranuclear palsy (PSP), atypical Parkinsonian syndrome (APS), principles of echo shifting with a train of observations (PRESTO), statistical parametric mapping (SPM)

## Abstract

**Purpose:**

Various magnetic resonance imaging (MRI) techniques including T_2_*-weighted imaging, susceptibility-weighted imaging, and MR relaxometry had been performed to evaluate different patterns of brain iron depositions in Parkinsonian syndrome. The aim of the present study was to evaluate the diagnostic value of a volume of interest (VOI) analysis on the principles of echo shifting with a train of observations (PRESTO) imaging using the statistical parametric mapping (SPM) 8 and the WFU PickAtlas program for the diagnosis of Parkinsonian syndrome.

**Methods:**

Fifty subjects, including 13 with the Parkinsonian variant of multiple system atrophy (MSA-P), 12 with progressive supranuclear palsy (PSP), 12 with Parkinson’s disease (PD) and 13 controls were evaluated in this study. After the spatial normalization of PRESTO images on SPM8, the WFU PickAtlas program was performed to create target VOIs in the putamen, red nucleus, substantia nigra, subthalamic nucleus, and dentate nucleus. The signal intensity ratio (SIR) was calculated by normalizing the signal of each VOI to that of the cerebrospinal fluid space. These SIRs were used as determinants in receiver operating characteristic (ROC) analyses.

**Results:**

SIR of the putamen was significantly lower in MSA-P than in PSP (*P* = 0.0051) and controls (*P* = 0.0004). In contrast, SIR of the red nucleus was significantly lower in PSP than in MSA-P (*P* = 0.0003), PD (*P* = 0.0029), and controls (*P* = 0.0011). In ROC analyses, SIR of the putamen exhibited the highest areas under the curves (AUCs) of 0.83 (vs. PSP) and 0.91 (vs. controls) in the diagnosis of MSA-P. On the other hand, SIR of the red nucleus exhibited the highest AUCs of 0.87 (vs. MSA-P), 0.90 (vs. PD), and 0.89 (vs. controls) in the diagnosis of PSP.

**Conclusions:**

The VOI analysis based on spatially normalized PRESTO images may be useful for depicting hypointensity, indicative of abnormal iron depositions, of the putamen and red nucleus in the diagnosis of MSA-P and PSP.

## Introduction

One of the common clinical issues encountered in the management of Parkinsonian disorders is the differentiation of Parkinson’s disease (PD) from atypical Parkinsonian syndromes (APSs) including the Parkinsonian variant of multiple system atrophy (MSA-P) and progressive supranuclear palsy (PSP). Correct diagnoses at early disease stages are important for adequate management. Brain MR imaging is recommended in the diagnostic work-up of Parkinsonism because it supports the diagnosis of APS or vascular Parkinsonism.^1^ It is also useful for diagnosing the other causes of Parkinsonism such as normal pressure hydrocephalus and brain tumors.

Recent studies have suggested that the sensitivity of long echo time high-resolution three-dimensional (3D) gradient echo sequences, including the principles of echo shifting with a train of observations (PRESTO) and susceptibility-weighted imaging (SWI), to brain iron depositions is greater than that of conventional T_2_*-weighted images (T_2_*WIs).^2,3^ These sequences also create better anatomic images on which basal ganglia structures are easily visible. Based on the different patterns of abnormal brain iron depositions between PD and APS, evaluating these disorders on PRESTO and SWI is considered appropriate. Previous studies utilizing PRESTO and SWI for the differentiation of Parkinsonism reported that these sequences provided new diagnostic markers for clinical use.^2,4–6^ These studies mainly conducted visual or manual region of interest (ROI) assessments. However, these methods are subjective and inadequate for the evaluation of anatomical structures, such as the subthalamic nucleus, which are difficult to identify on visual evaluation. Therefore, the aim of the present study was to evaluate the utility of an advanced objective analysis on PRESTO imaging for the differentiation of APS from PD.

## Materials and Methods

### Patients and control subjects

This was a retrospective study that evaluated the diagnostic value of an objective analysis on PRESTO from the data source at our medical center. This study utilized data obtained at a single medical center, and was approved by our Institutional Review Board. The privacy of the patients was completely protected.

In this study, the patient group was selected following a search of the medical records filed in our institution between August 2007 and December 2014. Patient backgrounds were standardized by applying the following inclusion criteria: (1) clinical diagnoses at the Department of Neurology according to the published criteria of PD, MSA-P, and PSP^7–9^ and (2) the acquisition of PRESTO images. An exclusion criterion was the insufficient quality of PRESTO images due to significantly abnormal findings (e.g., large cerebral infarctions and hemorrhages) adjacent to the volume of interest (VOI) described below and apparent artifacts that disturbed the spatial normalization process. Twelve PD (mean age, 65 ± 8 years; 3 men and 9 women), 13 MSA-P (mean age, 64 ± 8 years; 5 men and 8 women), and 12 PSP (mean age, 70 ± 5 years; 6 men and 6 women) patients were enrolled in this study. All PSP and MSA-P patients with full clinical data and follow-up information were included, whereas a subset of patients was selected randomly for PD in the alphabetical order of their names. Since patient samples with an unequal size are known to influence diagnostic values such as accuracy and area under the receiver operating characteristic (ROC) curve,^10,11^ the numbers of MSA-P, PSP, and PD patients were adjusted to be almost equal in the present study. Thirteen age-matched individuals (mean age, 63 ± 13 years; 5 men and 8 women) without obvious neurological and MR abnormalities were identified during the same period and enrolled as control subjects.

### MRI protocol

All 50 subjects underwent MRI examinations on a 1.5T imager (Gyroscan Intera; Philips Medical Systems, Best, The Netherlands) with a sensitivity-encoding parallel imaging head coil. Three-dimensional (3D) sections from PRESTO images were obtained in a transverse plane, for which the scanning parameters were as follows: repetition time (TR), 24–36 ms; echo time (TE), 34–46 ms; flip angle (FA), 20°; field of view (FOV), 230 mm; matrix, 320 × 320; and 2-mm-thick gapless sections. Additionally, 3D T_1_-turbo field echo (3DT_1_TFE) images were obtained in a sagittal plane, for which the scanning parameters were as follows: TR, 8.1–8.3 ms; TE, 3.7–3.8 ms; FA, 7°; FOV, 256 mm; matrix, 256 × 256; and 1-mm-thick gapless sections. All images were visually inspected for apparent artifacts such as patient motions.

## PRESTO Image Analysis

### PRESTO template creation and spatial normalization

To perform the automated analyses, it was necessary to create the template for the spatial normalization of PRESTO images. For this procedure, nine normal subjects (unpublished data set; mean age, 65 ± 10 years; 3 men and 6 women) who underwent both PRESTO and 3DT_1_TFE images were recruited. PRESTO and 3DT_1_TFE images of the nine normal subjects were initially converted from DICOM files to analyze formats using MRIcro (http://www.mccauslandcenter.sc.edu/mricro/mricro/) and transferred to statistical parametric mapping (SPM) 8 (Welcome Department of Cognitive Neurology, University College, London, UK), which was run on MATLAB version R2010a (The MathWorks Inc., Natick, MA, USA). For each subject, 3DT_1_TFE images were coregistered with PRESTO images. Then, a linear and nonlinear transformation of the 3DT_1_TFE images was performed. Pixel threshold was set to 10% of the whole-brain mean to eliminate background noises and partial volume effects at the edge of the brain. Data were standardized with the Montreal Neurological Institute (MNI) atlas using a 12-parameter affine transformation, followed by 16 nonlinear transformations and a trilinear interpolation. Spatially normalized images were not modulated (i.e., preserve concentrations). The final image format was 16-bit, with a size of 91 × 109 × 91 and a voxel size of 2 × 2 × 2 mm. This voxel size was sufficiently small to evaluate the deep nuclei.^12–14^ These transformation parameters of 3DT_1_TFE images were applied to normalize the corresponding subjects’ PRESTO images. A template of the PRESTO image was obtained by calculating a mean image from normalized PRESTO images of these nine normal subjects.

Finally, PRESTO images of 12 PD, 13 MSA-P, 12 PSP patients and 13 control subjects were spatially normalized onto this in-house-made PRESTO template in the MNI space.

### VOI analysis

**Fig 1.**
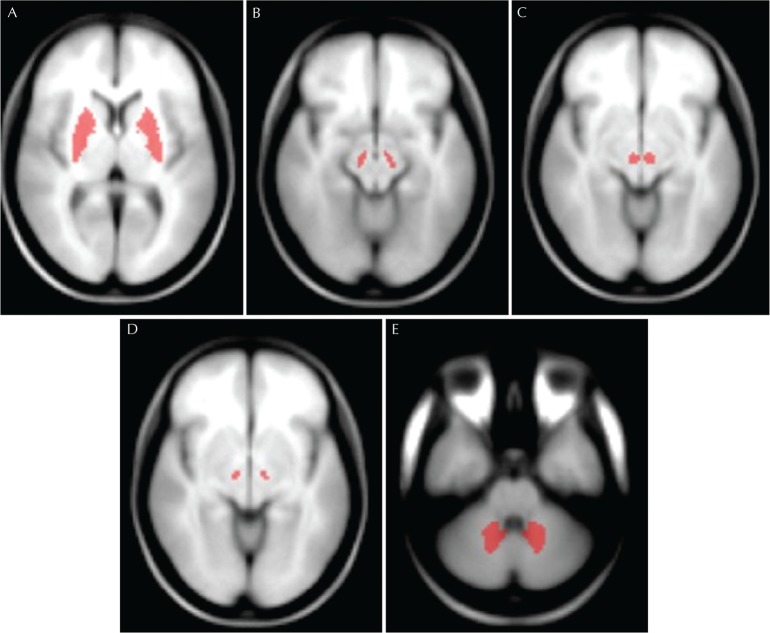
VOIs on a T_1_ template image of SPM8. VOIs are set in the bilateral putamen (**A**), substantia nigra (**B**), red nucleus (**C**), subthalamic nucleus (**D**), and dentate nucleus (**E**) according to anatomical structures in the Montreal Neurological Institute space. VOI, volume of interest.

VOIs were obtained with WFU PickAtlas (Talairach brain atlas theory), which automatically generated segmented atlas VOI templates in the MNI space.^15,16^ The VOIs defined in the atlas were originally based on manual delineation of the brain region borders according to the Talairach atlas. According to the previous studies that evaluated iron depositions in PD and APS,^2,4,5,17^ five bilateral hemispheric VOI labels provided as default settings by the software were evaluated: “putamen,” “substantia nigra,” “red nucleus,” “subthalamic nucleus,” and “dentate nucleus” (Fig. 1). The caudate nucleus and thalamus were not evaluated because these structures are known to be easily affected by atrophy and ventricular dilatation. The deposition of iron in these nuclei may be uneven and focal; therefore, the minimal signal intensity instead of the average of target VOIs was evaluated in order to avoid underestimation.^5^ Since PRESTO images were relative data, signal intensity normalization was performed by normalizing each VOI to that of the cerebrospinal fluid space.^18^ After spatial normalization and VOI placement, the presence of misregistration was visually evaluated.

### Statistical analysis

Statistical analyses were performed using IBM SPSS statistics 21 (IBM SPSS Inc., Chicago, IL, USA). The Kruskal-Wallis test for non-normally distributed data (gender, Hoehn-Yahr stage, disease duration, and signal intensity ratio (SIR) of the putamen, substantia nigra, subthalamic nucleus, and dentate nucleus), and a one-way analysis of variance for normally distributed data (age and SIR of the red nucleus) were performed for comparisons among patients and control groups. When a significant level was found in multiple comparisons, the Mann-Whitney U test or unpaired *t*-test was also performed. The relationships between SIR and clinical parameters including disease duration and severity were assessed by Spearman’s rank correlation coefficient or Pearson’s product-moment. After Bonferroni corrections for multiple comparisons, differences were considered significant when *P* < 0.0083. Using the SIR in target VOIs as a threshold, a ROC curve analysis was performed to determine which VOI discriminated the different groups. The Youden’s index was applied to determine the cut-off values.

## Results

Patient characteristics are summarized in Table 1. No significant difference was observed in age or sex among the patient and control groups. Furthermore, no significant differences were noted in the disease duration or severity (i.e., Hoehn-Yahr stage) between the patient groups.

**Fig 2.**
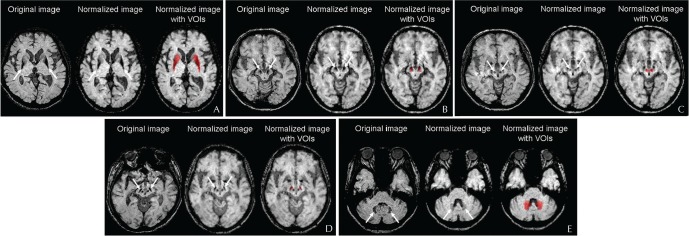
Typical original, normalized PRESTO images and VOIs of the putamen, substantia nigra, red nucleus, subthalamic nucleus, and dentate nucleus. Normalized PRESTO as well as original images reveal hypointensity of the bilateral putamen (**A**) in a 67-year-old female MSA-P patient, substantia nigra (**B**) and red nucleus (**C**) in a 67-year-old male PSP patient, subthalamic nucleus (**D**) in a 71-year-old male PSP patient, and dentate nucleus (**E**) in a 71-year-old female PSP patient (arrows). All original PRESTO and normalized images are displayed in the left and middle rows, respectively. Additionally, all normalized images with target VOIs are in the right rows. PRESTO, principles of echo shifting with a train of observations; VOI, volume of interest.

Typical original and normalized PRESTO images of the putamen, substantia nigra, red nucleus, subthalamic nucleus, and dentate nucleus are presented in Fig. 2. On visual inspection, there was no apparent misregistration of VOIs on spatially normalized PRESTO images in any patients or controls. The results of VOI analyses are shown in Table 2. SIR of the putamen was significantly lower in MSA-P patients (0.25 ± 0.15) than in PSP patients (0.52 ± 0.25; *P* = 0.0051) and controls (0.57 ± 0.18; *P* = 0.0004). In spite of the low putaminal SIR in MSA-P patients, no significant difference was observed between MSA-P and PD patients (0.25 ± 0.15 vs. 0.36 ± 0.18; *P* = 0.13). On the other hand, SIR of the red nucleus was significantly lower in PSP patients (0.55 ± 0.22) than in MSA-P patients (0.81 ± 0.15; *P* = 0.0003), PD patients (0.89 ± 0.12; *P* = 0.0029), and controls (0.84 ± 0.13; *P* = 0.0011). SIR in the substantia nigra and subthalamic nucleus were lower in PSP patients, but not significantly so. There were also no significant group differences in SIR of the dentate nucleus. A relationship was not found between clinical parameters and SIR of the putamen in MSA-P (duration; r = –0.27, *P* = 0.36 and severity; r = 0.05, *P* = 0.91) or that of the red nucleus in PSP (duration; r = –0.03, *P* = 0.93 and severity; r = –0.12, *P* = 0.70).

**Fig 3.**
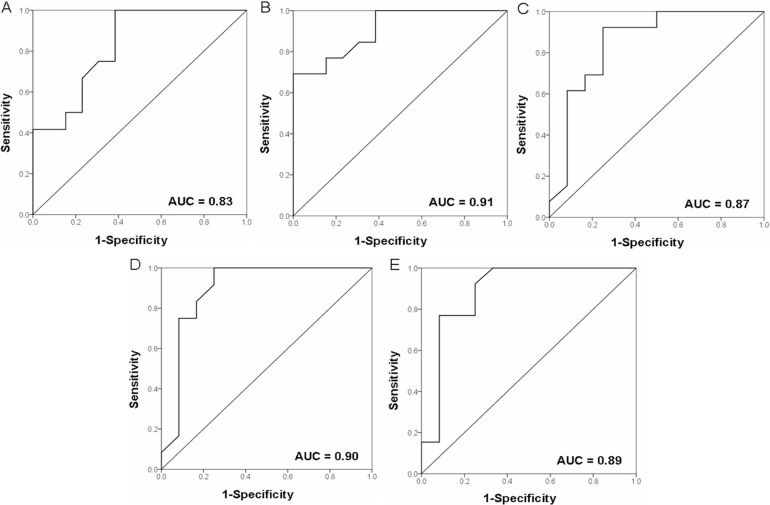
ROC curves for the differentiation of MSA-P from PSP (**A**) and controls (**B**) using SIR in target VOIs of the putamen, and for the differentiation of PSP from MSA-P (**C**), PD (**D**), and controls (**E**) using SIR in target VOIs of the red nucleus as a threshold. MSA-P, Parkinsonian variant of multiple system atrophy; PD, Parkinson’s disease; PSP, progressive supranuclear palsy; ROC, receiver operating characteristic; SIR, signal intensity ratio; VOI, volume of interest.

ROC analyses using SIR of the putamen and red nucleus to discriminate among MSA-P, PSP, and other groups were performed in order to evaluate the diagnostic accuracy of these target VOIs (Fig. 3). SIR of the putamen exhibited areas under the curve (AUC) of 0.83 and 0.91 for differentiating between MSA-P and PSP and between MSA-P and controls, respectively. SIR of the red nucleus exhibited AUC of 0.87, 0.90, and 0.89 for differentiating between PSP and MSA-P, PD, and controls, respectively. Cut-off values of SIR in these VOIs, and their sensitivities, specificities, and accuracies are shown in Table 3.

## Discussion

The results of the present study, which showed iron deposition in the putamen and red nucleus in MSA-P and PSP patients on PRESTO images, are concordant with previous findings.^2,4,5^ In these studies, PRESTO and SWI were mainly evaluated on visual inspection or manual ROI drawing. However, these subjective methodologies encompass various drawbacks including the inconsistency of inter-rater reliability.^4^ It is not considered suitable to evaluate particular anatomical structures such as the substantia nigra and subthalamic nucleus, which are difficult to recognize on visual inspection, using these methods. Although only a few studies have performed SPM to evaluate PRESTO and SWI,^19,20^ the voxel-based analysis (VBA) has some limitations. First, VBA is prone to errors caused by misregistration of anatomical structures. Due to the high sensitivity to susceptibility changes, particular structures including the base of the frontal lobes close to the skull base may be affected by the distortion, which can cause the misregistration, on PRESTO imaging. Second, compared with the VOI analysis, there are no standard conventions for what *P* value and multiple comparison correction to apply to statistical analyses in VBA. This can cause both Type 1 and 2 errors as a result of multiple comparisons, and potentially change the final conclusion of the study. On the other hand, the VOI analysis can select target structures which are not affected by distortions. Additionally, the VOI analysis requires less stringent statistical correction than VBA. Finally, the minimal signal intensity instead of the average of target VOIs was evaluated to overcome the disadvantage of the automated VOI analysis which caused false negative results by the inadequate VOI size.^21^ As a result, the combination of SPM and VOI analyses enables the objective evaluation of the substantia nigra and subthalamic nucleus, as performed in the present study. The limitation of deformation accuracy needs to be considered carefully for the interpretation of brain mapping analysis in atrophied brains. It is reported that MRI-aided spatial normalization is more accurate than the one performed by using only functional images, given the better anatomical information and higher spatial resolution of MR images.^22^ Therefore, MRI-aided normalization based on the coregistration of the 3DT_1_TFE and PRESTO images was performed to improve the accuracy of spatial normalization.^23^

PD and APS have been associated with the increased deposition of iron in pathoanatomically relevant structures. Increased concentrations of iron have primarily been reported in the putamen in postmortem studies of MSA-P patients.^24^ Furthermore, the red nucleus is an invariable structure of pathology in PSP, but is not involved in PD or MSA.^25,26^ The hypointensities of the putamen in MSA-P and the red nucleus in PSP on PRESTO imaging are consistent with previously reported neuropathological changes. However, the signal intensity of the substantia nigra is a debatable issue. In contrast to pathological studies indicating increased iron concentrations in the substantia nigra of PD,^17,24^ the present results as well as previous findings did not reveal apparent hypointensity in PD patients relative to APS patients or control subjects.^4,6^ Although there is no doubt about the increase of iron in the substantia nigra of PD, there are large discrepancies in the quantitative levels, ranging from 25% to 100% of the amounts normally observed.^24^ Of note, only ferritin iron is known to cause reduction of T_2_ relaxation and signal in a field-dependent manner, and pathological studies on iron levels of the substantia nigra indicate decreased ferritin levels and increased free iron levels in PD.^17^ These diversities of iron in the substantia nigra of PD can result in the conflicting results of signal intensity on MRI. Additionally, various factors including not only the deposition of iron, but also scan parameters may influence signal intensities on PRESTO and SWI. Previous studies used different 1.5T or 3T magnetic field strength imagers, which may partly explain the discrepancies observed in the evaluation of the substantia nigra in PD patients.^4–6,27^ In contrast to PRESTO imaging, SWI acquires magnitude and phase information, which enables the evaluation of phase shifting. We assumed that these different factors affected the signal intensity of the substantia nigra in PD. By considering the pathological changes indicating that the subthalamic nucleus and substantia nigra are commonly affected structures in PSP patients,^28^ PSP groups were expected to have relatively lower intensities in these structures. Iron deposition in the putamen in PD, even though less than in MSA-P, may also affect signal intensities.^26^ The absence of a relationship between disease severity and iron deposition is consistent with the hypothesis that iron deposition may represent a secondary response rather than a primary event promoting the cascade of neurodegeneration.^17,24^

The relatively small number of patients is a limitation of the present study, and may have affected the diagnostic value of VOI analyses on PRESTO imaging. Based on the effects of the magnetic field strength on sensitivity to susceptibility changes,^29^ it is desirable to perform PRESTO imaging on higher magnetic field images than 1.5T images. Considering the relative value of signal intensity on PRESTO imaging, the SIR method was applied in the present study. However, combination of VBA and other more quantitative methods including phase information and relaxometry may give further perceptions for the evaluation of iron depositions in the diagnosis of Parkinsonian syndrome. Furthermore, our study may also have been limited by the absence of pathological diagnoses in all cases. Therefore, we considered it necessary to investigate more cases of APS and PD in order to further clarify the diagnostic value of VOI analyses on PRESTO imaging.

## Conclusion

Our VOI analyses combining PRESTO imaging, SPM8, and the WFU PickAtlas demonstrated the diagnostic value of hypointensity, indicative of abnormal iron deposition, in the putamen and red nucleus for diagnosing MSA-P and PSP. Thus, an automatic objective evaluation of PRESTO imaging may represent a diagnostic marker for MSA-P and PSP.

## Figures and Tables

**Table 1.** Patient and control characteristics

	MSA-P (n = 13)	PSP (n = 12)	PD (n = 12)	Control (n = 13)	*P* value
Age at MRI (years)	64 ± 8	70 ± 5	65 ± 8	63 ± 13	0.30^*^
Male/Female	5/8	6/6	3/9	5/8	0.67^**^
Disease duration at MRI (years)	3.8 ± 1.2 (1–6)	5.5 ± 3.2 (2–13)	5.3 ± 2.8 (2–10)	NA	0.55^**^
Hoehn-Yahr stage	3.9 ± 0.9 (3–5)	3.7 ± 0.9 (2–5)	3.3 ± 0.4 (3–4)	NA	0.19^**^

Data are shown as absolute numbers or the mean ± standard deviation. MRI, magnetic resonance imaging; MSA-P, Parkinsonian variant of multiple system atrophy; NA, not applicable; PD, Parkinson’s disease; PSP, progressive supranuclear palsy;

* , one-way analysis of variance;

** , Kruskal-Wallis test.

**Table 2.** Comparison of SIR in target VOIs

	MSA-P (n = 13)	PSP (n = 12)	PD (n = 12)	Control (n = 13)	*P* value
Putamen	0.25 ± 0.15^*,**^	0.52 ± 0.25	0.36 ± 0.18	0.57 ± 0.18	0.0011^§^
Substantia nigra	0.76 ± 0.17	0.59 ± 0.29	0.78 ± 0.17	0.79 ± 0.14	0.32^§^
Red nucleus	0.81 ± 0.15	0.55 ± 0.22^#, ##, ###^	0.89 ± 0.12	0.84 ± 0.13	<0.0001^§§^
Subthalamic nucleus	0.74 ± 0.08	0.55 ± 0.19	0.70 ± 0.09	0.73 ± 0.10	0.10^§^
Dentate nucleus	0.67 ± 0.17	0.68 ± 0.17	0.71 ± 0.18	0.80 ± 0.14	0.11^§^

Data are shown as absolute numbers or the mean ± standard deviation. MSA-P, Parkinsonian variant of multiple system atrophy; PD, Parkinson’s disease; PSP, progressive supranuclear palsy;

§ , Kruskal-Wallis test;

§§ , one-way analysis of variance;

* *P* = 0.0051 vs. PSP by the Mann-Whitney U test;

** *P* = 0.0004 vs. controls by the Mann-Whitney U test;

# *P* = 0.0003 vs. MSA-P by the unpaired *t*-test;

## *P* = 0.0029 vs. PD by the unpaired *t*-test;

### *P* = 0.0011 vs. controls by the unpaired *t*-test. Because of Bonferroni’s multiple group comparisons, differences were considered significant when *P* < 0.0083.

**Table 3.** AUC, cut-off values, and diagnostic index for discriminating among MSA-P patients, PSP patients, and other groups

		AUC	CV	%								

				Sensitivity	Specificity	PPV	NPV	Accuracy
Putamen	MSA-P vs. PSP	0.83	0.23	100	62	100	71	82
	MSA-P vs. control	0.91	0.56	69	100	76	100	85
Red nucleus	PSP vs. MSA-P	0.87	0.67	92	75	90	80	83
	PSP vs. PD	0.90	0.68	100	75	100	80	88
	PSP vs. control	0.89	0.78	77	92	79	91	85

AUC, area under the curve; CV, cut-off value; MSA-P, Parkinsonian variant of multiple system atrophy; NPV, negative predictive value; PD, Parkinson’s disease; PPV, positive predictive value; PSP, progressive supranuclear palsy.
